# Ethereal Solution of Nitrate of Silver

**Published:** 1855-03

**Authors:** 


Etheral Solution of Nitrate of Silver.—Every one who has tried it is aware that the application of a watery solution of the nitrate of silver over a large extent of skin is a troublesome and patience-requiring process. The fluid does not dry quickly, and it runs about, being prevented, by the greasiness of the skin, from being rapidly absorbed. A plan which we see adopted by Mr. Ward, in the London Hospital, obviates very completely these difficulties. It consists in making the solution with the common nitric ether, instead of water. The ether acts as a solvent of any sebaceous matter which may be on the skin, and, from its volatility, very quickly dries in, producing, at the same time, a sensation of coolness very agreeable to the patient. If wished, several coatings may be applied successively to the same part, with loss of but little time. The strength which Mr. Ward generally employs is eight grains to the ounce; but it may, of course, be modified according to the wishes of the surgeon. The use of nitrate of silver externally, in erysipelas and other low forms of inflammation of the skin, is a very favorite practice in most of the London hospitals—either an aqueous solution, or the solid stick moistened, being usually employed. Mr. Ward informed us that the use of the ethereal menstruum was not original, but had been suggested to him by a gentleman by whom he had been consulted.—*Med. Times and Gaz.*

